# Possible Risk Factors and Their Potential Associations with Combined Heavy Metal Exposures in Pregnant Women in the Republic of Suriname

**DOI:** 10.5334/aogh.4402

**Published:** 2024-04-09

**Authors:** Vinoj H. Sewberath Misser, Ashna D. Hindori-Mohangoo, Arti Shankar, Maureen Lichtveld, Jeffrey Wickliffe, Dennis R. A. Mans

**Affiliations:** 1 Department of Pharmacology, Faculty of Medical Sciences, Anton de Kom University of Suriname, Paramaribo, Suriname; 2 Foundation for Perinatal Interventions and Research in Suriname (Perisur), Paramaribo, Suriname; 3 Tulane University School of Public Health and Tropical Medicine, New Orleans (LA), USA; 4 School of Public Health, University of Pittsburgh, Pittsburgh (PA), USA; 5 Department of Environmental Health Sciences, School of Public Health, University of Alabama at Birmingham, Alabama (AL), USA

**Keywords:** Suriname, pregnant women, combined heavy metal exposure, educational level, income level, region of residence, public health

## Abstract

**Background:**

The exposure of pregnant women to multiple environmental pollutants may be more disadvantageous to birth outcomes when compared to single-compound contaminations.

**Objective:**

This study investigated the mixed exposures to mercury, manganese, or lead in 380 pregnant Surinamese women. The factors that might be associated with the heavy metal exposures and the relative risk of the potential factors to cause the mixed exposures were explored. The influencing factors of exposures to mixed contaminants assessed were living in Suriname’s rural regions, several parts of which are contaminated with heavy metals emitted from artisanal and small-scale gold mining and agricultural activities; the consumption of potentially contaminated foods; advanced maternal age; as well as a relatively low formal educational level and monthly household income.

**Methods:**

Descriptive statistics were used to calculate frequency distributions and χ2-contingency analyses to calculate associations and relative risks (RR) with 95% confidence intervals (CI).

**Findings:**

Blood levels of two or three of the heavy metals above public health limits were observed in 36% of the women. These women were more often residing in the rural regions, primarily consumed potentially contaminated food items, were 35 years or older, were lower educated, and more often had a lower household income. However, only living in the rural regions (RR = 1.48; 95% CI 1.23–1.77) and a low household income (RR = 1.38; 95% CI 1.15–1.66) significantly increased the risk of exposure exceeding levels of concern to two or three of the heavy metals (by 48% and 38%, respectively).

**Conclusion:**

More comprehensive pharmacological, ecological, and epidemiological studies about exposures to mixed heavy metal contaminations in pregnant women are warranted.

## Introduction

The detrimental impact of heavy metals on pregnant women, their unborn children, and newborns has been well-established [1]. For instance, elevated levels of mercury or manganese in the blood of pregnant women have been associated with lower birth weight [2,3], increased maternal blood levels of lead with a higher incidence of preterm birth [4], and too high maternal lead and arsenic blood levels with a low Apgar score [5,6]. Pregnant women are particularly at risk of exposure to these and other heavy metals in low- and middle-income countries, where policies, legislation, and monitoring of residues in agricultural and industrial substances are insufficient [7,8].

This is also a concern in the Republic of Suriname, an independent country located on the north-eastern coast of South America. Suriname’s leading sources of subsistence are oil drilling, gold mining, agriculture, fishery, and forestry [9], which are mainly carried out in the country’s rural-interior and rural-coastal regions [10,11,12]. These activities substantially contribute to the country’s gross domestic income [13] but are accompanied by the release into the environment of the toxic heavy metals mentioned above [14,15].

Mercury spillage primarily occurs in the country by its use in amalgamating and extracting gold in the widespread small-scale artisanal mining activities [16,17]. Environmental pollution by lead may be attributed to the emission of fumes from lead-containing gasoline and paint products, residues from lead-based plumbing, and the application of lead-containing cosmetics [18,19,20,21]. Exposure of humans and contamination of the environment with manganese may occur during the large-scale production of staple foods such as rice, plantains, and cassava because of the use of illegal and banned agricultural pesticides and herbicides that contain this compound [22]. These compounds are persistent in the environment, accumulate in soils and sediments, and can cause damage to humans or inflict harm after entering the food chain [23,24,25].

We recently conducted a study with 380 pregnant women in Suriname aimed to determine the effects of prenatal exposure to mercury, manganese, and lead on birth outcomes [26]. For that purpose, an association was sought between levels of heavy metals in the women’s blood samples and the occurrence of adverse birth outcomes [26]. The study found no statistically significant relationship between maternal blood levels of these heavy metals and stillbirths, preterm births, low birth weights, or low Apgar scores [26]. However, a considerable proportion of the women had heavy metal blood levels exceeding the reference values of public health concern: 40.5% had mercury levels ≥ 3.5 µg/L, 63.9% had manganese levels ≥ 13.0 µg/L, and 21.3% had lead levels ≥ 3.5 µg/dL [26]. These findings indicate the need for public health measures to safeguard pregnant Surinamese women and their newborns from the harmful effects of exposure to environmental heavy metals.

An important aspect of this issue that deserves more attention is the risk of the simultaneous exposure of pregnant Surinamese women and their unborn children to mercury, manganese, and lead. That exposure to such mixed contaminations may occur in Suriname is not inconceivable when considering their geo-chemo-biological pathways, including their airborne transportation, accumulation in soils and sediments, conversion into highly toxic derivatives, and entry into the food chain following absorption by fish, vegetables, and staple foods, and presence in freshwater resources [22,23,27,28]. Simultaneous exposure to multiple heavy metals may not only be more harmful compared to exposure to single metals but may also carry a greater risk for adverse birth outcomes. For example, the combination of arsenic, cadmium, mercury, manganese, and lead reportedly might increase the risk of congenital heart defects in newborns [29] and the occurrence of asthma in young children [30], and newborns whose mothers had been exposed to lead, mercury, and cadmium had increased blood pressure levels [31].

With this background, descriptive statistics were applied to assess the frequency of contaminations with mercury, manganese, and lead at levels exceeding those of public health concern in the group of pregnant women in Suriname mentioned above. The economic activities associated with the potential spilling of these compounds mainly take place in Suriname’s rural-coastal and rural-interior regions [10,11,12]; the heavy metals may mainly accumulate in certain species of fish, vegetables, and staple foods [23,25,28,32]; and age at delivery, level of education, and household have previously been associated with a higher risk for exposure to contaminant mixtures [33,34,35,36]. The distribution of these potential risk factors for the mixed contaminants was also assessed using descriptive statistics. Finally, bivariate and relative risk analyses were used to explore potential associations and calculate relative risk ratios between these factors and the mixed contaminations.

## Methods

### Study design and setting

In this study, a group of 380 pregnant Surinamese women who had previously been evaluated for a potential relationship between exposure to mercury, manganese, and lead and adverse birth outcomes [26] was evaluated for the occurrence of combined heavy metals exposures. These women are a subset of the Caribbean Consortium for Research in Environmental and Occupational Health (CCREOH) cohort of 1200 pregnant Surinamese women who had been enrolled during 1 December 2016 to 30 September 2019 in a longitudinal epidemiological study that aims to evaluate the impact of environmental contaminants on birth outcomes and neurodevelopment of their children [37]. The CCREOH study (also known as the MekiTamara Study) was ethically approved by the Institutional Review Board of the Ministry of Health of Suriname (protocol number VG 023-14) and the Institutional Review Board of Tulane University, New Orleans, LA, USA (protocol number 83 093). All participants voluntarily entered the study, agreed to provide a venous blood sample, and provided informed written consent after a thorough explanation of the study’s purpose by trained research personnel who documented and witnessed the process. Participants who were under the age of 18 years were assented after the guardian(s) provided consent.

### Classification of combined heavy metals exposure

During the first or second trimesters, whole blood samples were collected from study participants. Blood levels of three heavy metals (mercury, manganese, and lead) had previously been determined and were categorized as either “low” or “high” based on public health cut-off levels.[26] For mercury, blood levels < 3.5 µg/L were considered “low” and those ≥ 3.5 µg/L were considered “high” [38]. For manganese, blood levels < 13.0 µg/L were considered “low” and those ≥ 13.0 µg/L “high” [39]. Blood levels of lead were considered “low” if < 3.5 µg/dL or “high” if ≥ 3.5 µg/dL [40]. Based on these cut-off points, the occurrence of the various “high-low” heavy metal combinations was recorded for each participant and subsequently categorized into four groups of combined heavy metals exposure: all three low levels; two low levels, and one high level; one low level and two high levels; all three high levels.

### Maternal characteristics

Information on maternal characteristics was collected at enrollment in the study by trained recruiters through face-to-face interviews using encrypted iPads, following the CCREOH’s study protocol [37]. Maternal characteristics deemed relevant to the current study included the region of residence, consumption of certain foods, maternal age at delivery, educational level, and monthly household income.

The region of residence was first categorized as urban-coastal, rural-coastal, and rural-interior according to the classification of the Surinamese General Bureau of Statistics [9] and subsequently as urban versus rural region. Foods that might represent sources of heavy metal contamination were fish, leafy vegetables, as well as staple foods such as rice, plantains, and cassava [41,42]. Maternal age at delivery was categorized into age groups 16–34 years and 35 years and older; educational level as none or primary education, and secondary or tertiary education; and monthly household income as USD < 75 and ≥ USD 75. These variables were taken as proxies for the socioeconomic status of participants [43].

### Statistical analysis

Descriptive statistics were calculated for combinations of heavy metals exposure (Table 1) and maternal characteristics (Table 2). Contingency tables were constructed to explore associations between maternal characteristics and heavy metal combinations (Table 3). Associations between pairs of variables were evaluated with the χ2-test and were expressed as p-values applying Fisher’s exact test for sensitivity adjustment. Relative risks (RR) with 95% confidence intervals (CI) were calculated if associations were significant. P values < 0.05 were considered statistically significant. All analyses were done using the Statistical Package for Social Sciences (SPSS version 25 for Windows).

**Table 1:** Number of heavy metals above public health cut-off levels in the pregnant Surinamese women included in the current study, the heavy metal combinations, and the number of women with a blood metal level combination (n = 380).


NUMBER OF HEAVY METALS ABOVE PUBLIC HEALTH CUT-OFF LEVELS	HEAVY METAL COMBINATIONS	NUMBER OF WOMEN (% OF TOTAL)

0	Hg Low| Mn Low| Pb Low	70 (18.4%)

1	Hg High| Mn Low| Pb Low	37 (9.7%)

	Hg Low| Mn High| Pb Low	131 (34.5%)

	Hg Low| Mn Low| Pb High	6 (1.6%)

2	Hg High| Mn High| Pb Low	61 (16.1%)

	Hg High| Mn Low| Pb High	24 (6.3%)

	Hg Low| Mn High| Pb High	19 (5.0%)

3	Hg High| Mn High| Pb High	32 (8.4%)


**Table 2:** Distribution of maternal characteristics in the study population (n = 380).


CHARACTERISTICS OF WOMEN EXPOSED TO HEAVY METAL MIXTURES	NUMBER OF WOMEN (% OF TOTAL)

Residing in the urban region	251 (66.1%)

Residing in the rural (coastal and interior) region	129 (33.9%)

Consumption of fish, leafy vegetables, and three or more staple food	356 (94.9%)

Aged 16–34 years	322 (84.7%)

Aged 35 years and older	58 (15.3%)

Primary or no formal education	66 (17.5%)

Secondary or tertiary education	312 (82.5%)

Household income < USD 75	112 (31.2%)

Household income ≥ USD 75	247 (68.8%)


**Table 3:** Associations between maternal characteristics and heavy metals combination (N = 380).


CHARACTERISTICS	BLOOD METAL COMBINATIONS				χ2- TEST RESULT*

	0 ≥ PUBLIC HEALTH LEVEL	1 ≥ PUBLIC HEALTH LEVEL	2 OR 3 ≥ PUBLIC HEALTH LEVEL	TOTAL	

	NUMBER (%)	NUMBER (%)	NUMBER (%)	NUMBER (%)	

**Region of Residence**					

Urban	57 (22.7%)	121 (48.2%)	73 (29.1%)	251 (100%)	**17.681**, **p < 0.001**

Rural (coastal and interior)	13 (10.1%)	53 (41.1%)	63 (48.8%)	129 (100%)	

**Dietary habits**					

Consumption of fish, leafy vegetables, and 3 or more types of staple food	66 (21.0%)	161 (45.2%)	129 (36.2%)	356 (100%)	0.300, p = 0.908

Else	4 (18.5%)	9 (47.4%)	6 (31.6%)	19 (100%)	

**Maternal age at delivery**					

16–34 years	57(17.7%)	153(47.5%)	112 (34.8%)	322 (100%)	2.696, p = 0.253

35 years and older	13 (22.4%)	21 (36.2%)	24 (41.4%)	58 (100%)	

**Educational level**					

Primary or no education	1(1.5%)	17(25.8%)	48(72.7%)	66(100%)	**50.579**, **p < 0.001**

Secondary or Tertiary	69(22.1%)	156(50.0%)	87(27.9%)	312(100%)	

**Household income(USD)**					

<75	13(11.6%)	42(37.5%)	57(50.9%)	112(10%)	**15.348,** **p < 0.001**

> = 75	51(20.6%)	123(49.8%)	73(29.6%)	247(100%)	


* Fisher’s exact test, p < 0.05, is considered significant.

## Results

### Distribution of heavy metal contaminations

Table 1 presents the prevalence of heavy metal combinations in the study population. High blood levels of all three heavy metals were found in 32 of the 380 participants (8.4%). More than one-quarter (104 of 380; 27.3%) had two heavy metals at high levels in their blood, and slightly less than half of the participants (174 of 380; 45.8%) had one heavy metal at high levels in their blood. Only 70 participants (18.4%) had low levels of all three heavy metals. Thus, more than one-third of the women examined (136 of 380; 35.8%) had concentrations of two or three heavy metals in their blood that surpassed public health cut-off points.

### Maternal characteristics

As shown in Table 2, the majority of participants (66.1%) were from Suriname’s urban-coastal region, but 129 (or about one-third) were from either the rural-coastal or the rural-interior region. Furthermore, the diet of 94.9% of the women included fish, leafy vegetables, and at least three types of staple food. About 15.3% of the participants were aged above 34 years, while 17.5% had no or only primary education, and less than one-third (31.2%) had a monthly household income of less than USD 75. Thus, approximately one-third of the women lived in a region with potential heavy metal pollution, virtually all consumed diets that could have been contaminated with heavy metals, the majority was in the mid-reproductive age, and most had a relatively high socio-economic status.

### Associations between maternal characteristics and heavy metal combinations in blood

In order to assess potential associations between maternal characteristics and mercury, manganese, and lead blood metal combinations, bivariate analyses were conducted. The results from these analyses are presented in Table 3. Region of residence, educational level, and monthly household income were statistically significantly associated with heavy metal combinations (p < 0.001), while no statistically significant associations were found for maternal age at delivery (p = 0.253) and the consumption of fish, leafy vegetables, and staple foods (p = 0.908) with heavy metal combinations in blood.

Low levels of all three heavy metals were statistically significantly more often noted in residents of the urban region when compared to those of the rural regions (22.7% vs. 10.1%; p < 0.001), in women who had a secondary or a tertiary education when compared to those with only primary education or who had no formal education at all (22.1% vs. 1.5%; p < 0.001), and in women who had a household income of 75 USD or more when compared to those with an income less than 75 USD (20.6% vs. 11.6%; p < 0.001).

Conversely, two or three high levels of mercury, manganese, or lead were statistically significantly more often observed in women residing in the rural regions when compared to those living in the urban region (48.8% vs. 29.1%; p < 0.001), women who had only a primary education or no formal education at all when compared to those with a higher education (72.7% vs. 27.9%; p < 0.001), and in women with a household income of less than 75 USD when compared to those with a higher income (50.9% vs. 29.6%; p < 0.001).

Next, the statistically significant associations observed in the previous paragraph were further explored by calculating the RRs by comparing two or three high metal combinations against no high metal combinations. Education level was excluded from this calculation as the data did not meet the sample size criteria. As shown in Table 4, living in the rural (coastal and interior) regions (RR = 1.48; 95% CI 1.23–1.77) and having a lower household income (RR = 1.38, 95% CI 1.15–1.66), increased the risk of exposure to two or three high heavy metals during pregnancy with 48% and 38% respectively. Thus, the risks of having a combination of two or three blood heavy metals above the public health cutoff points are statistically significantly higher (48–38%) in women living in the rural (interior and coastal) regions and in women with a low household income.

**Table 4:** Relative risk (95% CI)) of risk factors (region of residence, and household income) for 2 or 3 blood metal combinations above public health levels in pregnant women.


DETERMINANTS	BLOOD METAL COMBINATIONS				TOTAL	RELATIVE RISK (95% CI)	*χ*2- TEST RESULT*

	2 OR 3 ≥ PUBLIC HEALTH LEVEL		0 ≥ PUBLIC HEALTH LEVEL				
	
	NUMBER	%	NUMBER	%			

**Region of residence**							

Rural (coastal and interior)	63	82.9%	13	17.1%	76	1.48 (1.23 – 1.77)	**17.691, p < 0.001**

Urban	73	56.2%	57	43.8%	130	1 (reference)	

**Household income(USD)**							

<75	57	81.4%	13	18.6%	70	1.38 (1.15 – 1.66)	**15.348, p < 0.001**

> = 75	73	58.9%	51	41.1%	124	1 (reference)	


* Fisher’s exact test, p < 0.05, is considered significant.

## Discussion

The exposure of pregnant women to combinations of heavy metals may present a greater health risk for adverse birth outcomes when compared to single-agent contaminations [44,45], requiring increased public health vigilance and public health measures. The current study has assessed the distribution of combined contaminations with mercury, manganese, and lead in Suriname, the potential risk factors of the mixed contaminations (living in a specific part of Suriname, dietary habits, maternal age, and socio-economic status), as well as the relative risks of these factors to cause combined heavy metal exposures in a group of pregnant Surinamese women. Our results showed that more than one-third of the 380 women included in the study had two or three heavy metals in their blood at concentrations above public health levels of concern. Furthermore, a considerable proportion of the study population displayed the potential risk factors for mixed heavy metal exposures. Finally, living in Suriname’s rural areas, as well as having a low household income, might increase the risk for multiple exposures above levels of concern, whereas the consumption of fish, leafy vegetables, and staple foods, as well as older maternal age, did not.

The simultaneous exposure to two or three heavy metals at levels above recommended public health action levels—including mercury, manganese, and lead—has been reported before in, among others, Japan [46], the USA [47], and China [29]. In line with the results from the current study, the investigators suggested that education and low income might represent potential risk factors for exposure to multiple contaminants [46,48]. However, whereas these investigators also suggested that fish consumption and maternal age might represent potential risk factors, this did not seem to be the case in the current study.

The increased risk of exposure to multiple contaminants of pregnant women from the rural areas of Suriname with two or three heavy metals might be attributable to their exposure to the noxious emissions from artisanal gold mining and agricultural activities in particularly these parts of the country [49,50]. Indeed, the consumption of fish from polluted areas represented an important risk factor for multiple heavy metal intoxications in various parts of the world [46,47,48]. Furthermore, pesticide residues and heavy metal contaminations of soil and produce have been traced back to large-scale agricultural activities in various countries, including China [51]. The precise factors contributing to a higher risk of contaminations in the rural areas must be investigated in future studies.

Low education and low income (as well as other indicators of an unfavorable socioeconomic status) may also represent potential risk factors for multiple heavy metal exposures above public health levels of concern. This possibility has been reported in Puerto Rico [52] and Uruguay [53]. Low education and low income may limit access to and consciously choosing healthy and safe food [54,55] and may be associated with inadequate knowledge about the proper use of pesticides [56,57]. These characteristics have also been associated with the inability to recognize potentially contaminated dietary items [58], a greater risk of occupational exposure to toxic substances [59,60] and insufficient financial means to take proper protective measures [56,57].

Surprisingly, the consumption of potentially contaminated fish, leafy vegetables, and staple foods did not seem to carry a statistically significant risk for mixed exposure to mercury, manganese, and/or lead in the current study. Indeed, a previous study detected high mercury concentrations in blood and hair samples of inhabitants from villages in Suriname’s interior [61,62], whose main protein source is predatory fish that consume mercury-contaminated prey [23]. However, another report identified pesticide residues in vegetables that are widely consumed by pregnant Surinamese women that, fortunately, were below the cut-off points of the European Union [32]. This has recently been corroborated by Alcala and coworkers [63], who detected traces of pesticide metabolites in the urine of pregnant Surinamese women, possibly following contamination through food intake, dermal contact, or inhalation. It is important to determine whether urinary levels of heavy metals are also limited to traces rather than potentially dangerous concentrations in the women evaluated in the current study when considering that the majority of them consumed diets that potentially contained multiple heavy metals.

The absence of a statistically significant association between the older age of pregnant women and a higher risk of exposure to mixed heavy metal contaminations noted in the current study is not in agreement with the results from previous studies [46,47,48]. For instance, in a Chinese investigation that specifically explored the occurrence of heavy metals in different age groups, higher concentrations were found in middle-aged women when compared to younger women [64]. This might be attributable to the increasing susceptibility of the human body to environmental exposures with increasing age [65], particularly in individuals suffering from comorbidities such as hypertension [66]. The discrepancy between the data from the literature and those from the current study may be attributable to the relatively small size of the study population that, in addition, mainly consisted of women of reproductive age.

In summary, the results from the current study suggest that particularly women living in the rural areas of Suriname and women with a low household income were at risk for exposure to combined heavy metal contaminants. However, despite suggestions about the sources of the mixed heavy metal contaminants (particularly widespread artisanal gold mining and large-scale agriculture), the precise nature of the geo-chemo-biological pathways involved in the contaminations, and their combined pharmacological effects on humans, including pregnant women and their offspring are not clear [67,68]. The small sample size of the study population also makes it difficult to extrapolate the findings to the general population.

Nevertheless, the results from the current study emphasize the need for improved insights into exposure to mixed heavy metal contaminants. Notably, such exposures (including those with mercury, manganese, and/or lead) are also believed to be involved in the development of chronic ailments such as Parkinson’s disease, hypertension, and chronic kidney disease [66,69,70], presumably by perturbing metal homeostasis in the body and causing cell degeneration [71,72]. So far, studies on their effects on pregnant women, their unborn children, and their newborns are limited. Therefore, it is important to identify the precise geo-chemo-biological pathways involved in these phenomena and to understand their toxicokinetics and toxicodynamics better in order to implement the proper public health interventions.

## Conclusion

About one-third of the 380 pregnant Surinamese women evaluated in the current study had a combination of mercury, manganese, and/or lead in their blood at levels exceeding those of public health concern. Women living in the rural areas of Suriname and women who had a low household income ran a risk of 38–48% being exposed to one of these heavy metal combinations. More comprehensive pharmacological, ecological, and epidemiological studies about mixed heavy metal contaminations of pregnant women are warranted.

## Data Accessibility Statement

Data are available upon request after approval of the CREEOH research team and permissions of the ethical boards.
